# Supplementary material for: Mutations in PCYT2 disrupt etherlipid biosynthesis and cause a complex hereditary spastic paraplegia
Source: Brain. 2019 Oct 22;142(11):3382–97. doi: 10.1093/brain/awz291 (PMC6821184; doi:10.1093/brain/awz291)
Supplement: awz291_Supplementary_Data [file awz291_supplementary_data.zip › awz291-Suppl_data/Supplementary_Data4.zip!/Supplementary information 4 - plasma lipidomics.zip!/Plasma lipidomics/Control vs Patient2/Statistical plots and data/PCA_scores.pdf]

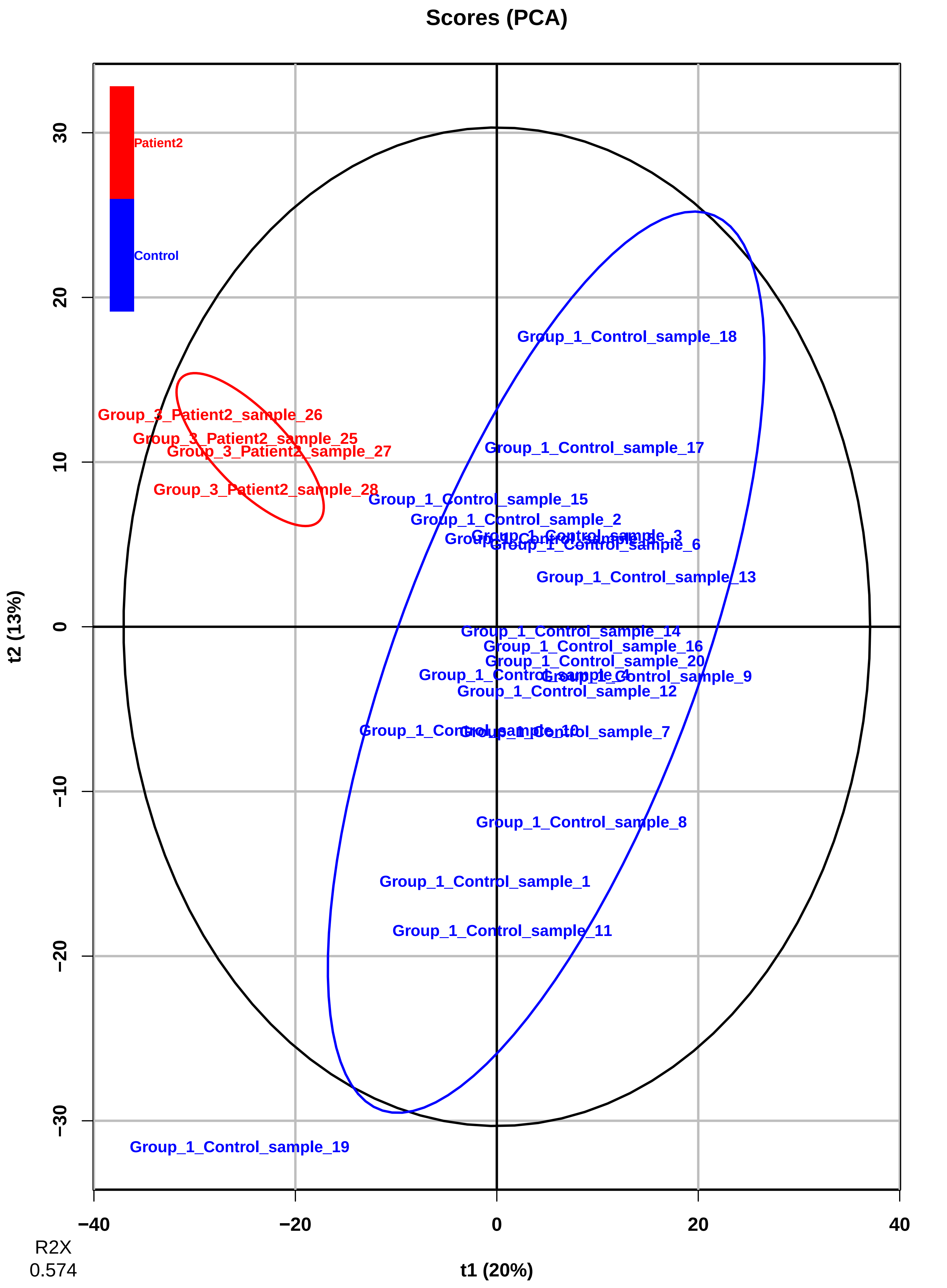
Scores (PCA)
Patient2
Control
Group_1_Control_sample_18
Group_3_Patient2_sample_26
Group_3_Patient2_sample_25
Group_3_Patient2_sample_27
Group_1_Control_sample_17
Group_3_Patient2_sample_28
Group_1_Control_sample_15
Group_1_Control_sample_2
Group_1_Control_sample_3
Group_1_Control_sample_6
Group_1_Control_sample_13
Group_1_Control_sample_14
Group_1_Control_sample_16
Group_1_Control_sample_20
Group_1_Control_sample_4
Group_1_Control_sample_9
Group_1_Control_sample_12
Group_1_Control_sample_10
Group_1_Control_sample_7
Group_1_Control_sample_8
Group_1_Control_sample_1
Group_1_Control_sample_11
Group_1_Control_sample_19
30
20
10
0
−10
−20
−30
t2 (13%)
−40
−20
0
20
40
t1 (20%)
R2X
0.574
